# Classification of temporomandibular joint hypermobility based on lateral TMJ, and magnetic resonance imaging contributing to a nonsurgical treatment protocol

**DOI:** 10.1038/s41598-026-36461-x

**Published:** 2026-02-12

**Authors:** Ayman F. Hegab, Mohammad Shuman, Hossam Abd Al Hameed, Khaled Karam

**Affiliations:** 1 Department of Oral and Maxillofacial Surgery, Faculty of Dental Medicine, Al-Azhar University in Cairo, Cairo, Egypt; 2 Department of Oral & Maxillofacial Surgery, Faculty of Dental Medicine, Al-Azhar University -Assiut, Assiut, Egypt; 3 Department of Diagnostic Radiology, Faculty of Medicine for Men, Al-Azhar University, Cairo, Egypt

**Keywords:** TMJ, Hypermobility, Condylar position, Disk position, Blood injection, I-PRF, Musculoskeletal system, Diseases, Medical research, Rheumatology

## Abstract

Hypermobility of the temporomandibular joint (TMJ) may present with a wide range of condylar and disk positions. The aim of this study was to evaluate condylar and disk positions in patients with varying degrees of TMJ hypermobility and to propose a new classification system with an associated nonsurgical treatment protocol. A consecutive sample of 144 patients with internal derangement was enrolled. Clinical examinations, lateral TMJ radiographs, and magnetic resonance imaging (MRI) were performed. Patients were classified according to the proposed staging system and treated with the corresponding nonsurgical protocol. The primary outcome variables were maximum voluntary mouth opening and visual analogue scale (VAS) pain scores. The secondary outcome variable was joint sounds. Statistical analysis demonstrated a significant decrease in mouth opening during the study period (P < 0.001 at 12 months posttreatment). VAS scores also showed a statistically significant reduction across all study groups and time points (P < 0.0001 at 12 months posttreatment). Joint sounds improved significantly throughout the study period (P < 0.001 at 12 months posttreatment). The proposed classification system is simple and practical, providing a detailed description of condylar and disk positions in TMJ hypermobility. The associated nonsurgical treatment protocol proved to be effective and tailored to the pathological changes observed in the joint.

## Introduction

Dislocation of the temporomandibular joint refers to the displacement of the condylar head from its normal position in the glenoid fossa of the squamotemporal portion of the cranial base. It may be partial (subluxation) or complete (luxation), bilateral or unilateral, and classified as acute, chronic protracted, or chronic recurrent^1–6^.

Dislocations may occur in anterior-medial, superior, medial, lateral, or posterior directions. The etiology can be spontaneous or associated with trauma, forceful mouth opening during endotracheal intubation with laryngeal mask or tracheal tube, ear-nose-throat or dental procedures, endoscopy, excessive yawning, laughing, vomiting, or seizures^7–10^.

Structurally altered factors predisposing individuals to TMJ dislocation include capsular laxity, weak ligaments, a small, short, or atrophic condyle, an atrophic or elongated articular eminence, a hypoplastic zygomatic arch, and a shallow glenoid fossa^7–11^.

Systemic predisposing conditions include epilepsy, severe vomiting, Ehlers-Danlos syndrome, Marfan syndrome, and dystonic movements secondary to major tranquilizers or neuroleptics prescribed for neuropsychiatric disorders^7–11^.

Condylar hypermobility is closely associated with TMJ structure and function, as well as the dynamics of the masticatory system. The joint capsule, reinforced by the lateral ligaments, plays a critical role in stabilization. However, the displacement of the condylar head from the glenoid fossa is also significantly influenced by the morphology of the condyle, glenoid fossa, and articular eminence^12–16^.

These factors primarily determine the type and direction of dislocation. In addition, age, dental status, etiology, duration of dislocation, and the functional state of the masticatory muscles contribute substantially to the mechanism and management of TMJ dislocation^17,18^.

Anterior dislocations are the most common type and occur when the condyle is displaced anterior to the articular eminence of the temporal bone. They usually result from an interruption in the normal sequence of muscle action during the mouth closure following extreme opening.

The masseter and temporalis muscles elevate the mandible before the lateral pterygoid muscle relaxes, causing the mandibular condyle to be pulled out of the glenoid fossa and positioned anterior to the bony eminence. Spasm of the masseter, temporalis, and pterygoid muscles produces trismus and prevents the condyle from returning to the glenoid fossa^19^.

TMJ hypermobility is usually identified only when it disrupts smooth mandibular movements. In such cases, clicking sounds at the end of opening or the beginning of closing, as well as jerky lateral mandibular movements, may be observed^20^.

These interferences may be caused by condylar dislocation during wide opening, occurring anterior and superior to the crest of the eminence. Alternatively, they may result from posterior displacement of the disc relative to the condyle. Snapping of the condyle over the crest of the eminence or the anterior band of the disc may also interfere with smooth mandibular movements.

Symptomatic hypermobility is typically not associated with complaints unless accompanied by myogenous pain secondary to dislocations, or when the condyle has such difficulty passing the crest of the eminence or the anterior band of the disc that proper mouth closure is impaired^21–25^.

Based on clinico-radiological evaluation, Akinbami classified TMJ dislocation into three types:

**Type I**: The head of the condyle lies directly below the tip of the eminence.

**Type II**: The head of the condyle lies anterior to the tip of the eminence.

**Type III**: The head of the condyle lies high and anterior to the base of the eminence^26^.

Patel et al. further classified hypermobility according to the condylar position relative to the eminence. A four-line axis was drawn perpendicular to the eminence, and the condylar position was traced to define five types of relations:

**Type 1:** The condyle is posterior and superior to the highest point of the eminence.

**Type 2:** The condyle is posterior to the eminence but at the same level as its highest point.

**Type 3:** The condyle is located directly below the highest point.

**Type 4:** The condyle is anterior to the eminence but at the same level as its highest point.

**Type 5:** The condyle is anterior and superior to the eminence^27^.

In 1832, Sir Astley Cooper proposed principles for the diagnosis and treatment of dislocation of the lower jaw. He introduced the terms complete dislocation (luxation) and imperfect dislocation (subluxation), which were later further delineated by other authors. Subluxation is generally defined as displacement of the condyle out of the glenoid fossa and anterosuperior to the articular eminence, which can be reduced by the patient (self-reduced). Clinical and radiographic analyses have shown that approximately 70% of the population can subluxate the TMJ. In contrast, dislocation is a similar displacement of the condyle that cannot be self-reduced^16^.

The stability of any joint depends on three factors: the integrity of the ligaments associated with the joint, the activity of the musculature acting on the joint, and the bony architecture of the joint surfaces. Treatments for recurrent dislocation may therefore be organized according to these stability factors: alteration of the ligaments, alteration of the associated musculature, and alteration of the bony anatomy^28^.

Evaluation of maximal mouth opening is frequently used as a clinical indicator of TMJ pathology. An increased maximal mouth opening is often considered evidence of articular hypermobility or a dislocated condylar head^29^.

This relationship has been strongly questioned by authors who analyzed the condylar position at maximum opening. Imaging at varying degrees of condylar excursion, without clinical signs of dislocation or subluxation, suggests that the position of the condyle at maximum mouth opening is not directly related to the magnitude of condylar movement^30–33^.

Overextension of the disc–condyle complex beyond the tip of the articular eminence during wide opening can predispose to disc displacement, with or without reduction. Recently, Hegab et al. described two distinct stages of disc displacement associated with joint hypermobility: Stage 2C and Stage 3C^34,35^.

Treatment of TMJ hypermobility varies in the literature, ranging from patient education to open surgical procedures. Conservative methods include intra-articular injection of sclerosing agents such as alcohol, sodium tetradecyl sulfate, sodium psylliate, sodium morrhuate, and platelet-rich plasma.

The use of autologous blood injection in recurrent dislocation, first reported by Brachmann in 1964, remains widely practiced. Another conservative approach is the application of botulinum toxin A (BTX-A) in recurrent TMJ dislocation^4,36,37^.

Surgical procedures aim either to remove mechanical obstacles in the condylar path or to create such obstacles by augmenting the articular eminence^38–41^.

The purpose of this study was to analyze condylar position at maximum mouth opening and the associated disc displacement using lateral TMJ radiographs and MRI in patients with hypermobility, with the goal of establishing a new classification system to guide nonsurgical treatment protocols.

## Patients and methods

*Ethics statement* The study complied with the tenets of the Declaration of Helsinki for research involving human participants and was reviewed and approved by the Institutional Review Board of the Al-Azhar University School of Dentistry. Written informed consent was obtained from all patients enrolled in the study.

## Patients and study design

### Classification strategy and patient assignment to stages

The classification strategy and research protocol followed the work of Hegab et al. ^34^ for establishing a classification system of TMJ internal derangement. To evaluate the validity of the new system, an independent retrospective cohort study was conducted. Records of 50 normal patients, including lateral TMJ images and MRI scans, were collected and analyzed.

**Fig. 1:**
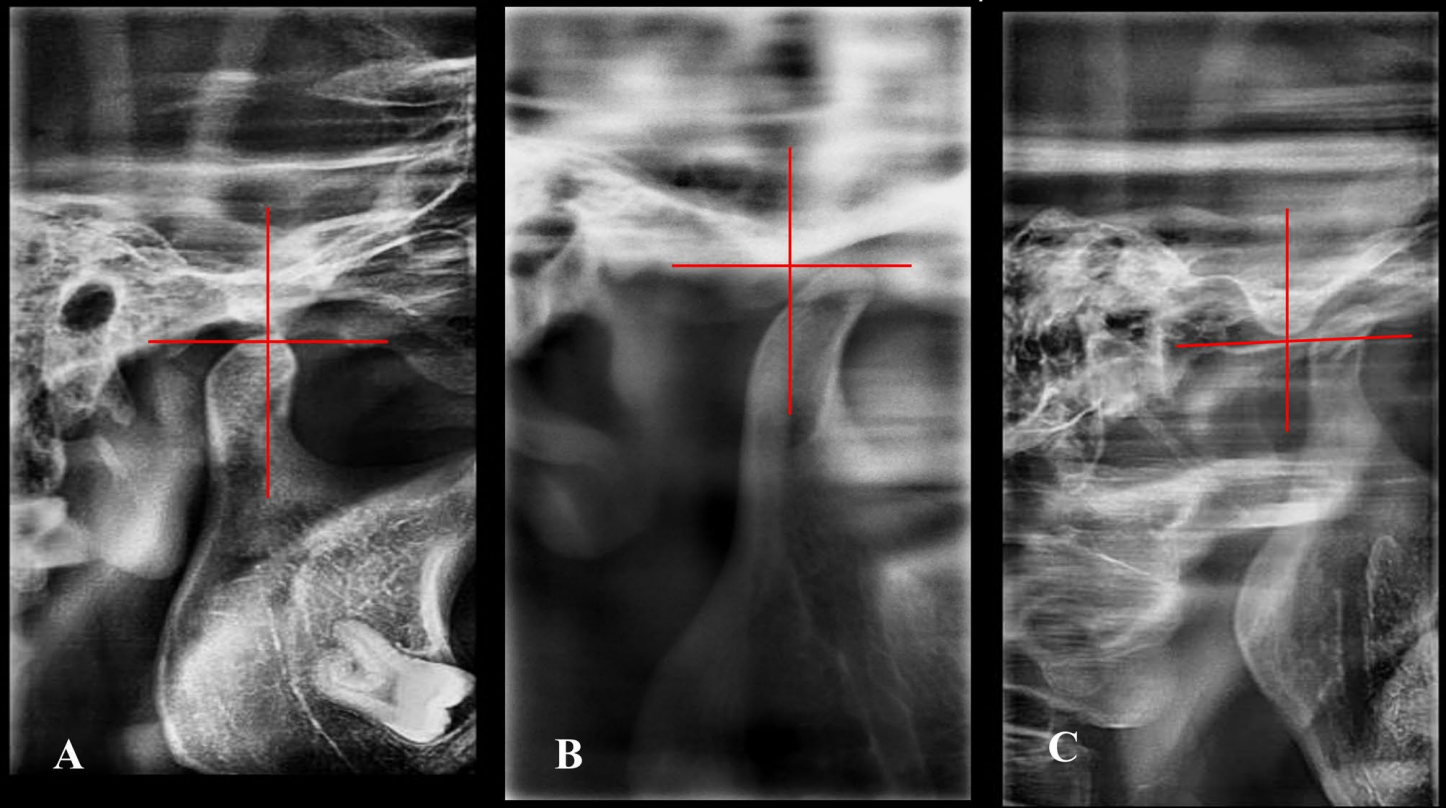
Flowchart representing the process of patient assignment into groups

In the present study, both lateral TMJ images and MRI scans were used to develop the new diagnostic classification system. Based on this system, therapeutic guidelines were proposed**. **The primary key point was the condylar position on the lateral TMJ view (Fig. 1**). **Secondary key points were the absence or presence of disk displacement and its direction (anterior vs posterior) on MRI.

**Fig. 2:**
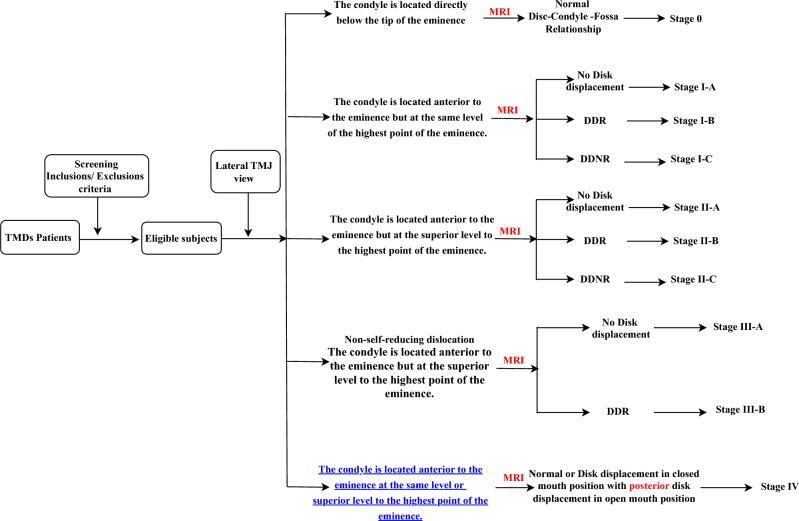
The relation of the condylar position to the articular eminence was evaluated on the lateral TMJ view at maximum mouth opening. **Position I:** The head of the condyle is directly below the tip of the eminence**. Position II:** The head of the condyle is in front of the tip of the eminence. **Position III**: The head of the condyle is high-up in front of the base of the eminence.

The primary key points were used for classification staging, whereas the secondary key points were applied for sub-staging (Fig. 2**)**. Flowcharts illustrate the process of patient assignment into groups. Each lateral TMJ image and MRI scan was evaluated independently by radiologists. To validate the new classification system, each radiologist independently assigned patients to a stage and sub-stage. Cases were classified into the correct stage only when both radiologists agreed. The purpose of sub-staging was to detect the presence or absence of TMJ disk displacement and, subsequently, to guide appropriate treatment.

**Fig. 3:**
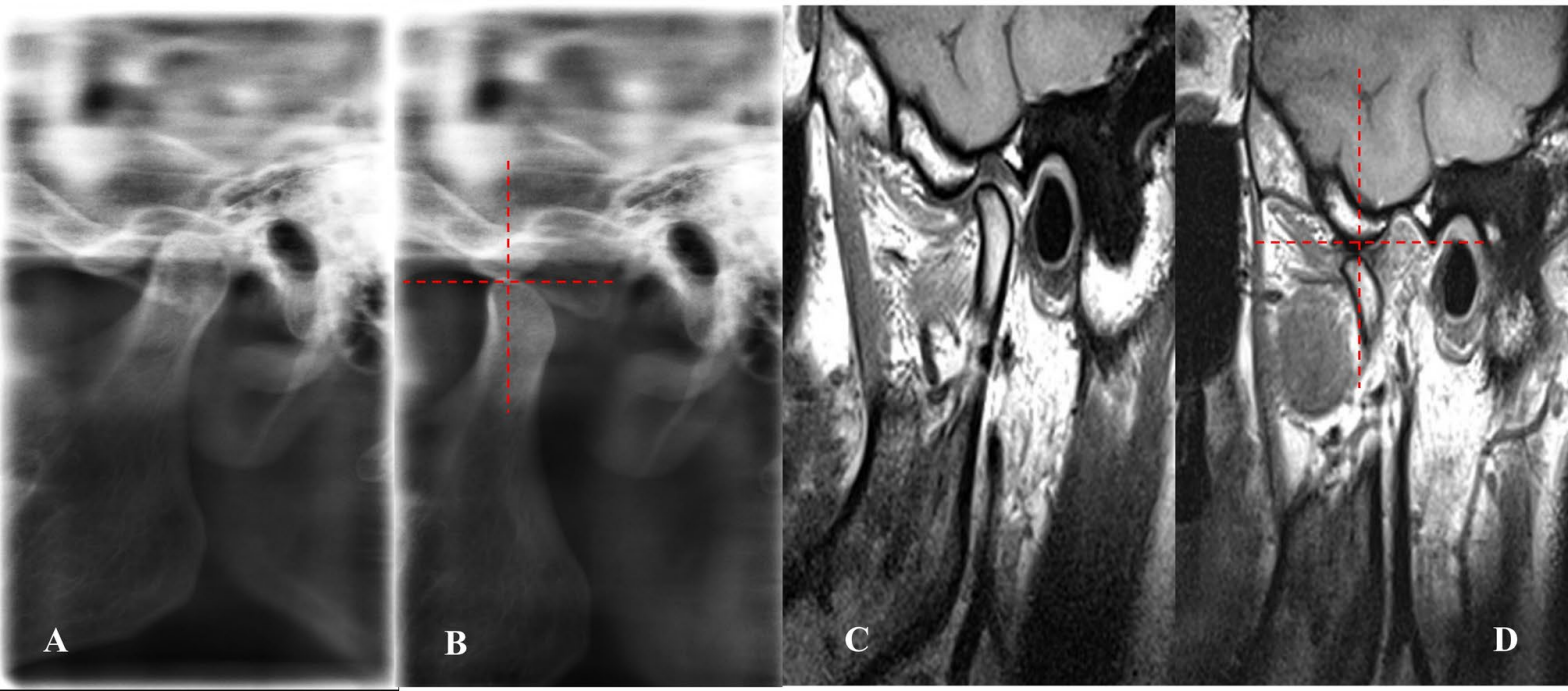
Lateral TMJ view representing **Stage 0.** Closed mouth **(A)** and open mouth **(B)** positions show the condyle located directly below the tip of the eminence. Oblique sagittal T1-weighted images representing **Stage 0** in closed mouth **(C)** and open mouth **(D)** position show a normal condyle-disk-fossa relationship. The disk-condyle complex is located directly below the tip of the eminence

The new classification system, based on lateral TMJ images and MRI, is divided into five stages:

*Stage 0 (Normal Mouth Opening)* The lateral TMJ image shows the condyle located directly below the tip of the eminence, with a normal disk-condyle-fossa relationship on MRI. (Fig. 3**).**

**Fig. 4:**
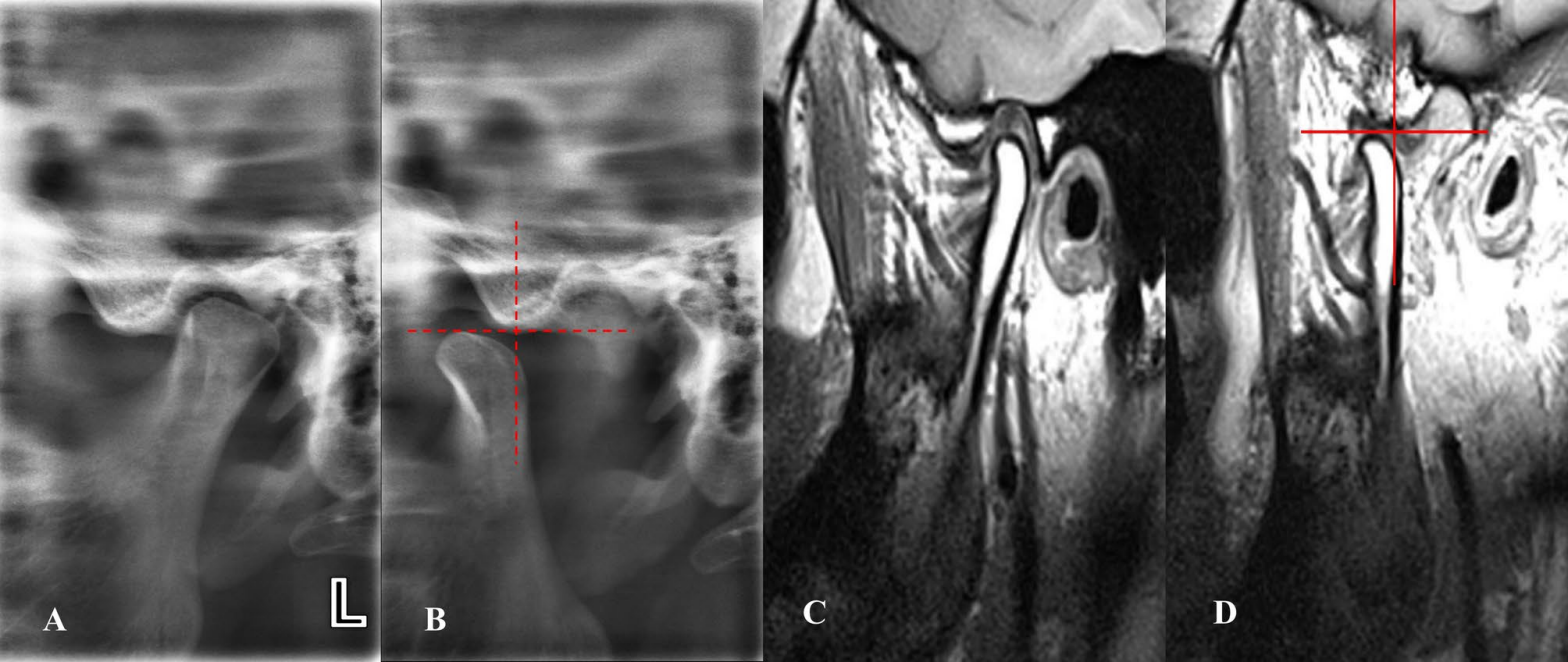
Lateral TMJ view representing Stage I-A. Closed mouth **(A)** and mouth **(B)** position show the condyle located anterior to the eminence but at the same level as the highest point of the eminence. Oblique sagittal T1-weighted images representing Stage I-A in closed mouth **(C)** and open mouth **(D) **positions show a normal condyle-disk-fossa relationship. The disk-condyle complex is located anterior to the eminence but at the same level as the highest point of the eminence

*Stage 1A (self-reducing subluxation)* The lateral TMJ image shows the condyle located anterior to the eminence but at the same level as its highest point. MRI is normal. The disk-condyle complex is anterior to the eminence but remains at the same level as its highest point (Fig. 4**).**

**Fig. 5:**
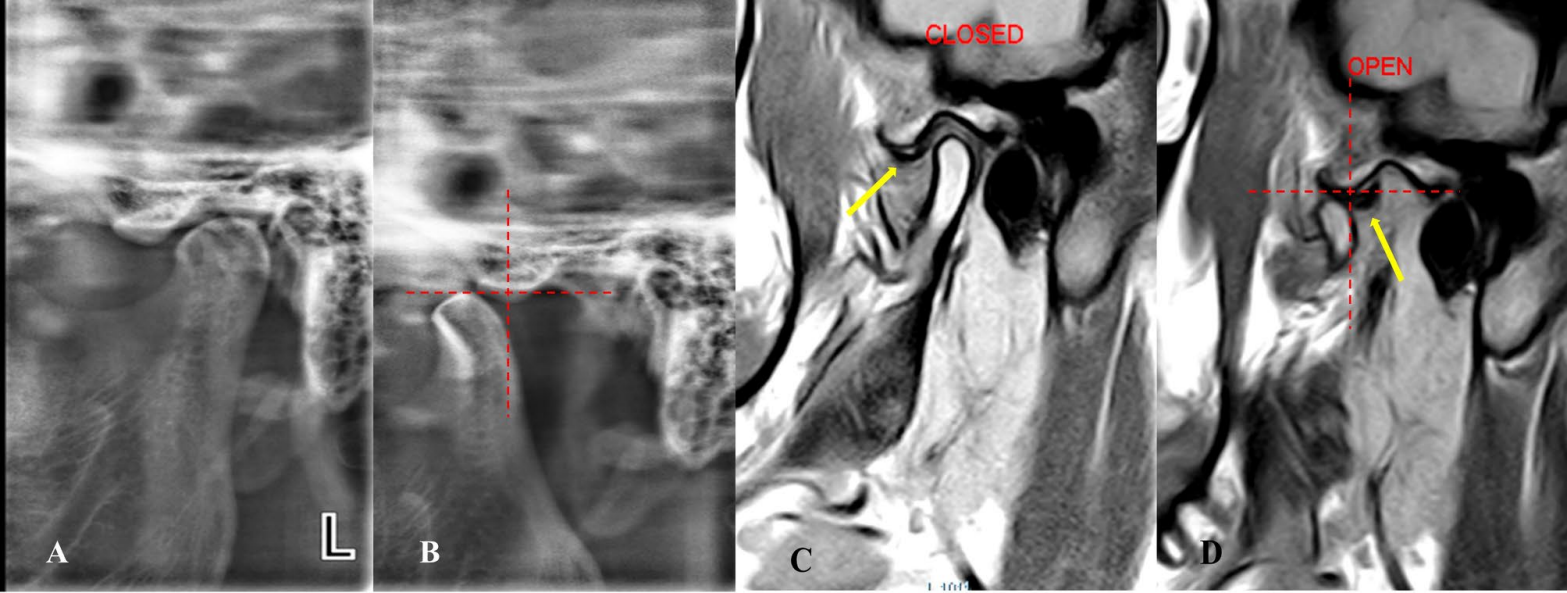
Lateral TMJ view represent Stage I-B in closed **(A)** mouth position & in open **(B)** mouth position showed the condyle is located anterior to the eminence but at the same level of the highest point of the eminence. Oblique sagittal T1-weighted images representing stage I-B showed anterior disk displacement (yellow arrow) in closed **(C)** mouth position with recapture of the articular disk (yellow arrow) during open **(D)** mouth position. The position and the disk-condyle complex position at the same level of the highest point of the eminence

*Stage 1B (self-reducing subluxation)* The lateral TMJ image shows the condyle located anterior to the eminence but at the same level as its highest point. MRI demonstrates disk displacement in the closed-mouth position with recapture of the articular disk during mouth opening. The disk-condyle complex remains at the same level as the highest point of the eminence (Fig. 5**).**

**Fig. 6:**
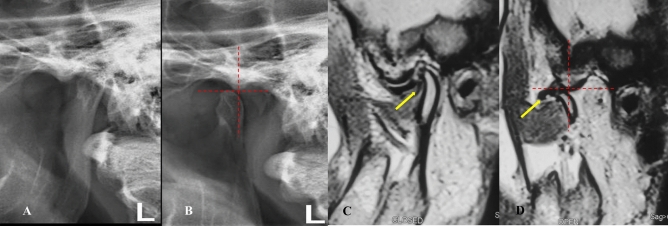
Lateral TMJ view represents stage I-C in closed **(A)** mouth position and in open **(B)** mouth position, showing the condyle located anterior to the eminence but at the same level as the highest point of the eminence. Oblique sagittal T1-weighted images represent Stage I-C, showing anterior disk displacement (yellow arrow) in closed **(C)** mouth position without recapture of the articular disk (yellow arrow) during open **(D)** mouth position. The disk-condyle complex remains at the same level as the highest point of the eminence

*Stage 1C (self-reducing subluxation)* The lateral TMJ image shows the condyle anterior to the eminence but at the same level as its highest point. MRI demonstrates disk displacement in the closed-mouth position without recapture during mouth opening. The disk-condyle complex remains at the same level as the highest point of the eminence (Fig. 6**).**

**Fig. 7:**
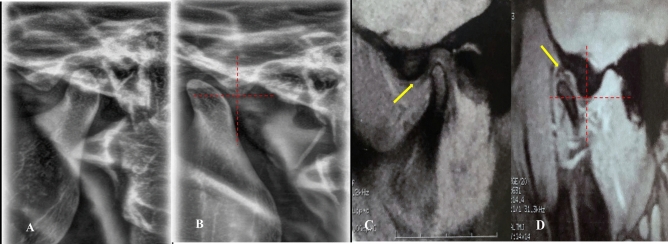
Lateral TMJ view represents stage II-A in closed **(A)** mouth position and in open **(B)** mouth position, showing the condyle located anterior to the eminence but at a superior level to the highest point of the eminence. Oblique sagittal T1-weighted images represent Stage II-A, showing a normal condyle-disk-fossa relationship (yellow arrow) in closed **(C)** mouth position and in open **(D)** mouth position, with the disk-condyle complex located anterior to the eminence but high up in front of the base of the eminence.

*Stage 2A (self-reducing subluxation)* The lateral TMJ image shows the condyle located anterior to the eminence but superior to its highest point. MRI is normal. The disk-condyle complex lies high and anterior to the base of the eminence (Fig. 7**).**

**Fig. 8:**
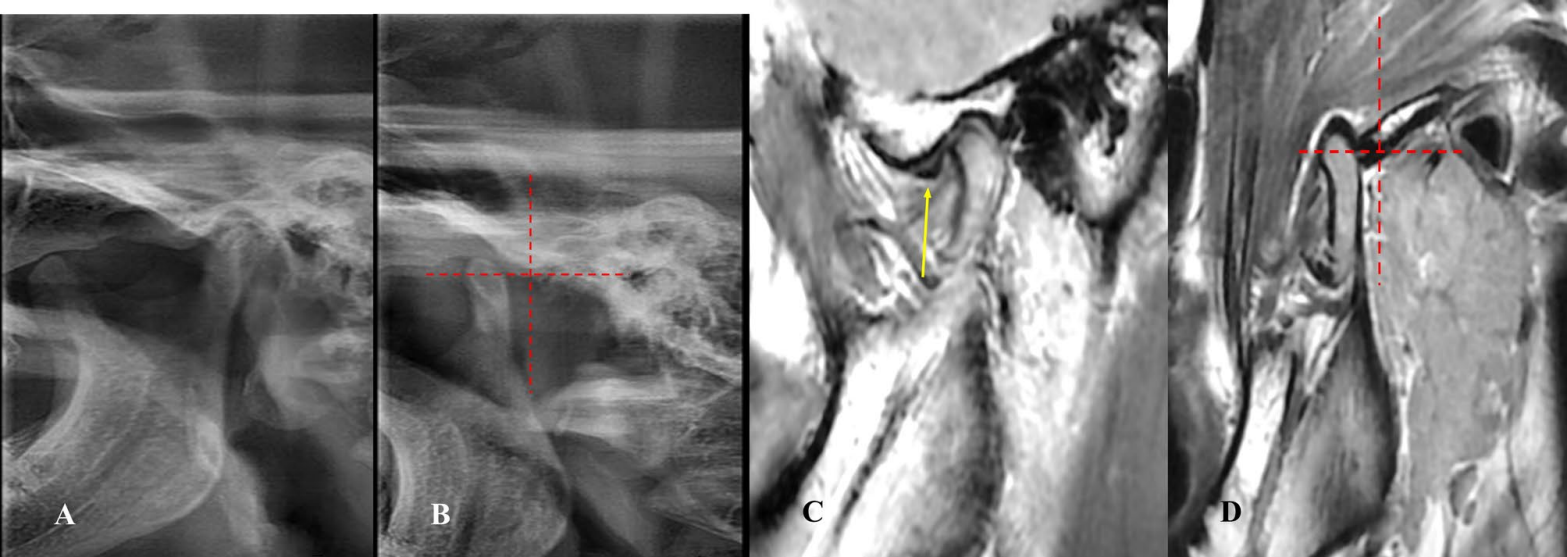
Lateral TMJ view represents stage II-B in closed **(A)** mouth position and in open **(B)** mouth position, showing the condyle located anterior to the eminence but at a superior level to the highest point of the eminence. Oblique sagittal T1-weighted images represent Stage II-B, showing anterior disk displacement (yellow arrow) in closed **(C)** mouth position with recapture of the articular disk (yellow arrow) during open **(D)** mouth position. The disk condyle complex positioned high up in front of the base of the eminence.

*Stage 2B (self-reducing subluxation)* The lateral TMJ image shows the condyle located anterior to the eminence but superior to its highest point. MRI demonstrates disk displacement in the closed-mouth position with recapture during mouth opening. The disk-condyle complex lies high and anterior to the base of the eminence (Fig. 8**).**

**Fig. 9:**
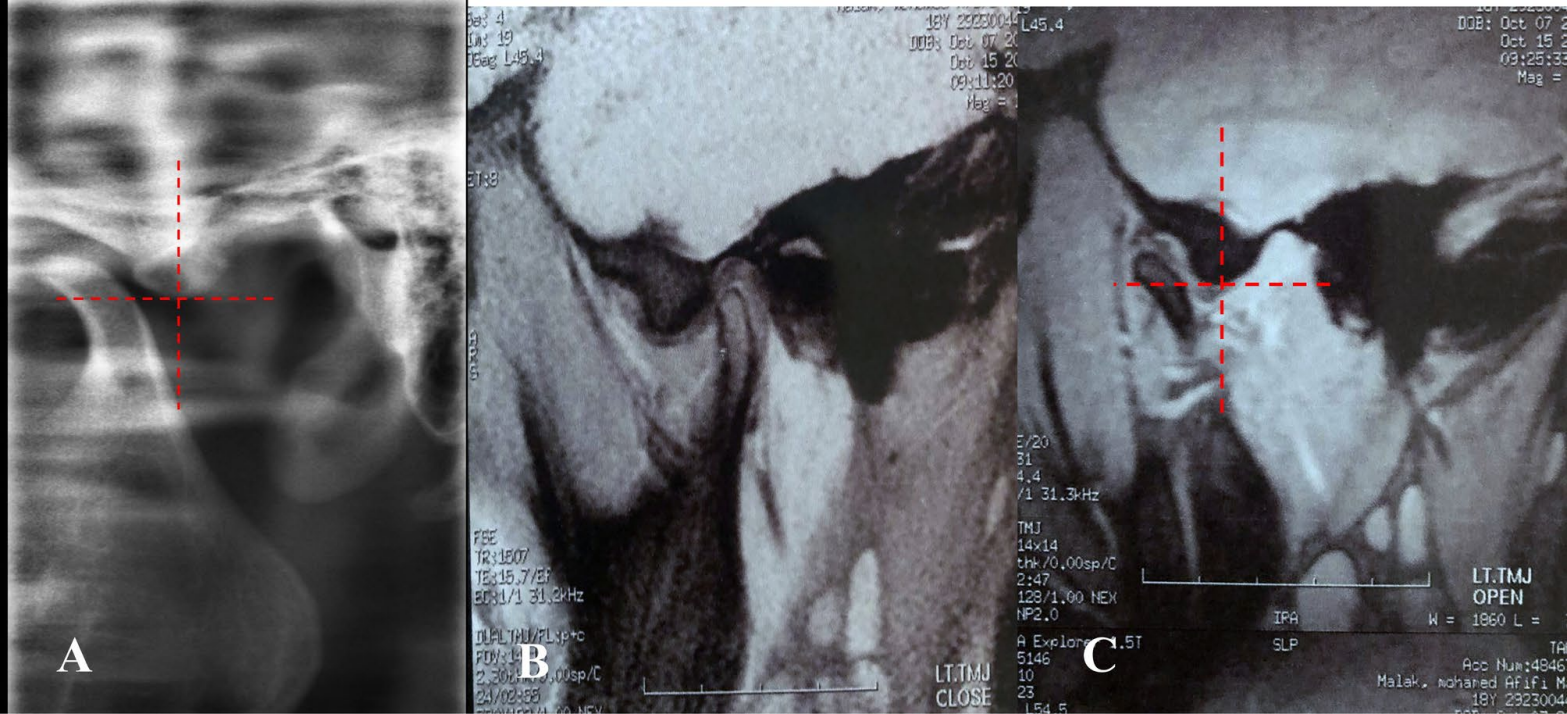
Lateral TMJ view represent Stage III-A (open lock) in open **(A)** mouth, showing the condyle located anterior to the eminence but at a superior level to the highest point of the eminence. Oblique sagittal T1-weighted images (done after reduction of the open lock) represent Stage III-A, showing a normal condyle-disk-fossa relationship in closed **(B)** mouth position and in open **(C)** mouth position. The disk-condyle complex is located in front of the tip of the eminence or high up in front of the base of the eminence.

*Stage 3A (non-self-reducing dislocation)* The lateral TMJ image shows the condyle located anterior to the eminence but at a superior level to its highest point. MRI is normal. The disk-condyle complex lies high and anterior to the base of the eminence **(**Fig. 9**).**

**Fig. 10:**
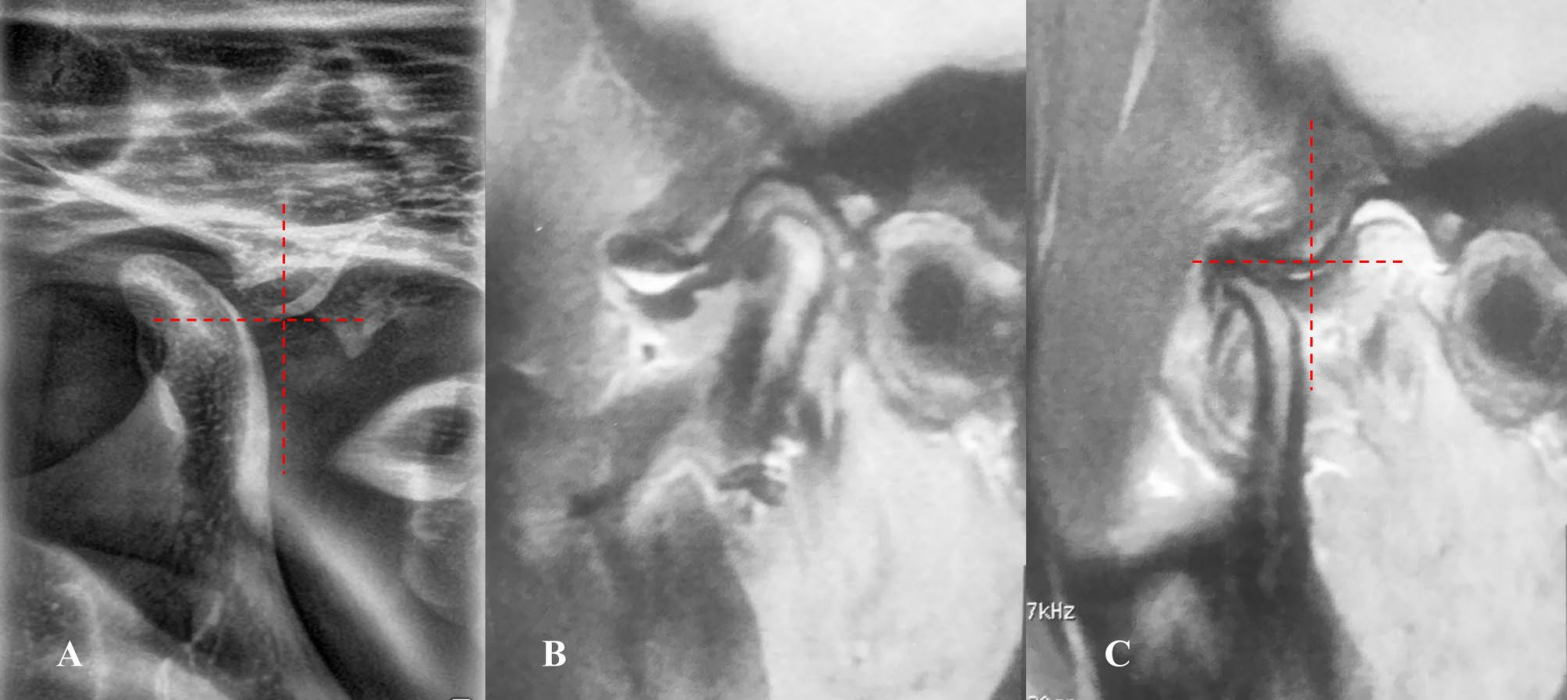
Lateral TMJ view represents Stage III-B (open lock) in open **(A)** mouth position, showing the condyle located anterior to the eminence but at a superior level to the highest point of the eminence. Oblique sagittal T2-weighted images (done after reduction of the open lock) represent Stage III-B, showing disk displacement in closed **(B)** mouth position and disk recapture in open **(C)** mouth position with joint effusion. The disk-condyle complex is located in front of the tip of the eminence or high up in front of the base of the eminence.

*Stage 3B (non-self-reducing dislocation)* The lateral TMJ image shows the condyle located anterior to the eminence but superior to its highest point. MRI demonstrates disk displacement in the closed-mouth position without recapture during mouth opening. The disk-condyle complex lies high and anterior to the base of the eminence (Fig. 10**).**

**Fig. 11:**
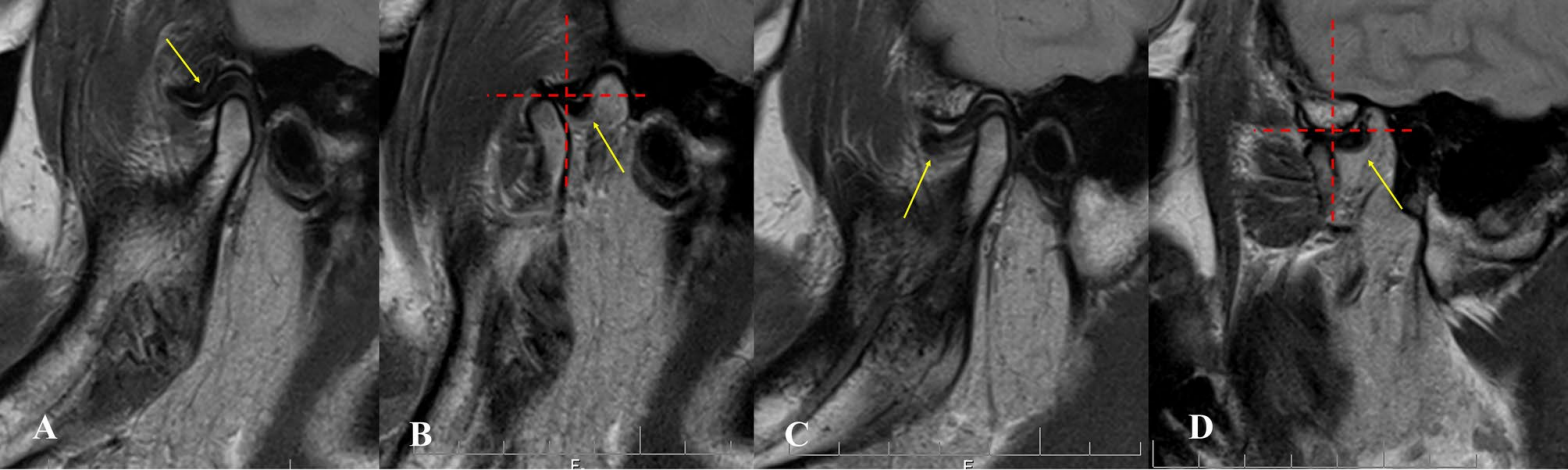
Oblique sagittal T1-weighted images represent Stage IV, showing a normal disk-condyle-fossa relationship (yellow arrow) in closed **(A)** mouth position with posterior disk displacement (yellow arrow) in open **(B)** mouth position. Oblique sagittal T1-weighted images represent Stage IV, showing anterior disk displacement (yellow arrow) in closed **(C)** mouth position with posterior disk displacement (yellow arrow) in open **(D)** mouth position.

*Stage 4 (self-reducing subluxation)* The lateral TMJ image shows the condyle located anterior to the eminence at the same or superior level relative to its highest point. MRI may show disk displacement in the closed-mouth position with posterior displacement during mouth opening (Fig. 11**).**

To address the research purpose, the investigators conducted a prospective cohort clinical study. The study sample included patients seeking treatment for TMD et al.-Azhar University Hospital and the outpatient clinic of the Faculty of Dental Medicine, Al-Azhar University. Eligible patients provided written consent and underwent treatment for TMJ disorders between February 2020 and February 2024.

### Inclusion criteria

Patients were eligible for inclusion if they had TMJ hypermobility confirmed by lateral TMJ views in both closed- and open-mouth positions. Chronic recurrent dislocation of the TMJ was diagnosed based on patient history and clinical findings.

### Exclusion criteria

Patients were excluded if they had undergone prior treatment for TMJ disorders (e.g., joint injection, splint therapy, or joint surgery). Additional exclusion criteria included systemic diseases such as rheumatoid arthritis, psoriatic arthritis, or juvenile arthritis, as well as unwillingness to participate.

### Clinical evaluations

Potential participants were screened by medical history and physical examination. A standardized protocol was followed for all participants. Maximum voluntary (non-assisted) mouth opening (MVMO, in millimeters), pain scores using a visual analogue scale (VAS), and joint sounds were recorded for all patients enrolled in the study.

### Radiographic evaluations

Lateral TMJ images were used to assess the relationship of the condylar position to the articular eminence at maximum range of motion. This was evaluated using landmarks described in Akinbami’s classification^20^ A four-line axis perpendicular to the eminence was drawn, and condylar position was defined as follows: Position I: The condylar head lies directly below the tip of the eminence**. **Position II**:** The condylar head lies anterior to the tip of the eminence. Position III: The condylar head lies high and anterior to the base of the eminence.

*MRI evaluation* MRI was used to assess the disk-condyle complex in relation to the articular eminence. For each TMJ, one sagittal slice through the middle of the long axis of the condyle was selected from both open- and closed-mouth positions for evaluation of the mandibular condyle and articular disc. Coronal views were not considered in this study. All MRI evaluations and measurements were performed using DICOM (Digital Imaging and Communication in Medicine) digitizing software.

Patients diagnosed clinically with TMJ hypermobility were evaluated by MRI to determine the presence or absence of disk displacement, with or without reduction. MRI scans were reviewed for all participants and categorized according to the Hegab classification. Patients with Hegab stages 2C and 3C were included in this study:

*Hegab stage 2C* MRI demonstrates anterior disk displacement in the closed-mouth position, with reduction to the normal position in the open-mouth position, accompanied by condylar hypertranslation.

*Hegab stage 3C* MRI demonstrates anterior disk displacement in the closed mouth position without reduction in the open mouth position. This stage is associated with normal or hypertranslation of the mandibular condyle and no limitation of mouth opening.

MRI scans were examined by two radiologists with more than 20 years of experience in TMJ imaging. The scans were assessed for the presence or absence of disk displacement. Normal disk position in the sagittal oblique plane was defined as the posterior band of the disk at the 12 o’clock position relative to the mandibular condyle in the closed-mouth position. In the open-mouth position, the thin intermediate band was located between the mandibular condyle and the articular eminence. Disk displacement with reduction (DDR): In the closed mouth position, the posterior band of the disk is anterior to the condyle; in the open mouth position, the disc returns to its normal position between the condyle and articular eminence. Disk displacement without reduction (DDNR): In the closed-mouth position, the posterior band of the disk is anterior to the condyle; in the open-mouth position, the posterior band remains anterior and does not return to its normal position between the condyle and articular eminence^34,42^. For patients in stages III-A and III-B (non-self-reducing dislocation), lateral TMJ views and open-mouth MRI were obtained first. After reduction of the dislocation, MRI scans were repeated in the closed-mouth position.

### Treatment protocol therapeutic tools

Patient education and functional modification include the following: habit awareness, avoidance of wide yawning, and avoidance of contact sports.

*Joint* *Injection *The main nonsurgical treatment tool was TMJ arthrocentesis followed by joint injection with blood (main injecting material) or injection of injectable platelet-rich fibrin (I-PRF) in cases of associated osteoarthritis/disk degeneration. This was followed by jaw immobilization with extraoral face-lift bandage, IMF screws, or orthodontic braces for 2 weeks.

*TMJ splint therapy (Hegab TMJ splint)* The splint is a hard, full-coverage maxillary occlusal splint with indentation. The splint was used by the patient all the time except during eating. The splint leads to adjustments of occlusion, enhancement of jaw muscle function, and new positioning of the disk–condyle relationship. The splint vertical thickness was 4 mm for DDR and 6 mm for DDNR cases, with at least 1 year of treatment^34,42^.

*Treatment allocation to the different groups* The first and main nonsurgical treatment modality was joint injection with blood or I-PRF, which was used in all the classification stages and substages for many reasons: the technique is safe and can be repeated without complications.

Allocation was based on the following radiographic parameters: patients with hypermobility associated with TMJ osteoarthritis were treated with I-PRF, while patients with joint hypermobility without osteoarthritis were treated with blood injection.

Functional modification was used for all the classification stages and substages because it aimed to limit mandibular movement and subsequent joint subluxation or dislocation.

*Hegab TMJ splint (HTS)* was used in the classification stages and substages associated with disk displacement with or without reduction. In addition to its placebo effect, it leads to adjustments of occlusion, enhancement of jaw muscle function, and new positioning of the disk–condyle relationship. Functional modification was used for all the classification stages and substages because it aimed to decrease the joint load by decreasing unnecessary and traumatic joint movements.

Table 1 shows a detailed description of the new classification stages associated with the nonsurgical treatment protocol used in this study.

**Table 1:** Showed detailed description of the new classification system.

Stage	Clinical finding	Lateral TMJ finding	MRI finding	Treatment
Stage 0	Normal mouth opening	The condyle is located directly below the tip of the eminence (normal position at maximum mouth opening)	No Disk displacement in closed mouth position The disk-condyle complex located directly below the tip of the eminence	No treatment required Patient education
Stage I-A	Self-reducing subluxation	The condyle is located anterior to the eminence but at the same level of the highest point of the eminence	No Disk displacement in closed mouth position The disk-condyle complex is located anterior to the eminence but at the same level of the highest point of the eminence	No treatment required Patient education
Stage I-B	Self-reducing subluxation	The condyle is located anterior to the eminence but at the same level of the highest point of the eminence	Disk displacement in closed mouth position with recapture of the articular disk during open mouth. The position and the disk-condyle complex position at the same level of the highest point of the eminence	⁶ Blood injection ⁶ or I-PRF injection in case of associated osteoarthritis/ disk degeneration ⁶ jaw immobilization by Extraoral Face-Lift bandage, IMF screws, or orthodontic braces for 2 weeks ⁶ HTS splint (**4mm**) for one year
Stage I-C	Self-reducing subluxation	The condyle is located anterior to the eminence but at the same level of the highest point of the eminence	Disk displacement in closed mouth position without recapture of the articular disk during open mouth position and the disk-condyle complex at the same level of the highest point of the eminence	⁶ Blood injection ⁶ or I-PRF injection in case of associated osteoarthritis/ disk degeneration ⁶ jaw immobilization by Extraoral Face-Lift bandage, IMF screws, or orthodontic braces for 2 weeks ⁶ HTS splint (**6mm**) for one year
Stage II-A	Self-reducing subluxation	The condyle is located anterior to the eminence but at the superior level to the highest point of the eminence	The disk-condyle complex position is high up in front of the base of the eminence No Disk displacement in closed mouth position	⁶ Blood injection ⁶ or I-PRF injection in case of associated osteoarthritis/ disk degeneration ⁶ jaw immobilization by Extraoral Face-Lift bandage, IMF screws, or orthodontic braces for 2 weeks
Stage II-B	Self-reducing subluxation	The condyle is located anterior to the eminence but at the superior level to the highest point of the eminence	Disk displacement in closed mouth position with recapture of the articular disk during open mouth position and the disk-condyle complex position is high up in front of the base of the eminence	⁶ Blood ⁶ or I-PRF injection in case of associated osteoarthritis/ disk degeneration ⁶ jaw immobilization by Extraoral Face-Lift bandage, IMF screws, or orthodontic braces for 2 weeks ⁶ HTS splint (**4mm**) for one year
Stage III-A	Non-self-reducing dislocation	The condyle is located anterior to the eminence but at the superior level to the highest point of the eminence	the disk-condyle complex in front of the tip of the eminence or high up in front of the base of the eminence. No Disk displacement in closed mouth position	⁶ Blood injection ⁶ or I-PRF injection in case of associated osteoarthritis/ disk degeneration ⁶ jaw immobilization by Extraoral Face-Lift bandage, IMF screws, or orthodontic braces for 2 weeks
Stage III-B	Non-self-reducing dislocation	The condyle is located anterior to the eminence but at the superior level to the highest point of the eminence	the disk-condyle complex in front of the tip of the eminence or high up in front of the base of the eminence with Disk displacement in closed mouth position with recapture of the articular disk during open mouth position	⁶ Blood injection ⁶ or I-PRF injection in case of associated osteoarthritis/ disk degeneration ⁶ jaw immobilization by Extraoral Face-Lift bandage, IMF screws, or orthodontic braces for 2 weeks ⁶ HTS splint (4mm) for one year
Stage IV	Self-reducing subluxation	The condyle is located anterior to the eminence at the same level or superior level to the highest point of the eminence	The disk-condyle complex position is high up in front of the base of the eminence With or without Disk displacement in closed mouth position with posterior disk displacement in open mouth position	⁶ Blood injection ⁶ or I-PRF injection in case of associated osteoarthritis/ disk degeneration ⁶ jaw immobilization by Extraoral Face-Lift bandage, IMF screws, or orthodontic braces for 2 weeks ⁶ HTS splint **(4-6mm)** for one year

Bold values represent vertical thickness of the occlusal splint is the key of hegab TMJ splint

### Evaluating the outcomes of the treatment protocol

The primary outcome variable was treatment effectiveness, assessed by comparing pretreatment and posttreatment MVMO (in millimeters). Pain scores were also recorded pretreatment and posttreatment using a 10-point visual analogue scale (VAS), with 0 indicating no pain and 10 indicating the worst pain.

The secondary outcome variable was joint sound. To evaluate joint sound before and after treatment, patients were asked to open their mouths as widely as possible. Joint sound was then assessed using three methods: (1) palpation of the TMJ zone by the clinician, (2) the patient’s self-report, and (3) auscultation of the TMJ zone with a stethoscope.

Absence of joint sound was confirmed when no sound was detected or reported by any of the three methods. Joint sound was considered present if it was detected or reported by one or more methods, or if the result was undetermined.

All outcome variables were assessed and compared within groups at baseline (pretreatment) and at 1, 3, 6, and 12 months posttreatment. Age and sex were considered additional variables and correlated with treatment outcomes.

Adjustment variables included baseline MVMO and baseline pain index scores. For statistical purposes, VAS pain levels and jaw range-of-motion values were treated as continuous variables. Repeated-measures analysis of variance (ANOVA) was performed to assess significant within-group and between-group treatment effects.

Adjustments for age and sex were included to assess the influence of demographic factors on treatment effectiveness.

### Statistical analysis

MedCalc version 12.3.0.0. was used to determine the study’s power (https://www.medcalc.org/en/download/) . The calculated power was 0.97 (97%), indicating that the sample size was adequate. Sample size calculation was performed using G*Power version 3.1.9.2.

Numerical data were explored for normality by examining distributions, calculating means and medians, and applying normality tests (Kolmogorov–Smirnov and Shapiro–Wilk tests).

Age data exhibited a parametric distribution, whereas inter-incisal opening data and VAS scores exhibited nonparametric distributions.

Age data are presented as mean ± standard deviation (SD) and were compared using Student’s t-test. Non-parametric data are presented as median and range values. The Mann–Whitney U test was used for between-group comparisons. The Friedman test was applied to evaluate changes within each group over time, and Wilcoxon signed-rank tests were used for pairwise comparisons when the Friedman tests showed significance.

Joint sound data (qualitative) are presented as frequencies (n) and percentages (%). The significance level was set at 0.05. All analyses were conducted using InStat statistical software (GraphPad Software, Inc., La Jolla, CA- https://www.graphpad.com/)^42^.

## Results

### Results of the retrospective cohort study

Interobserver reliability (95% confidence interval) scores between radiologists for MRI examinations were 0.97, indicating an excellent level of correlation. All 50 cases were assigned to the correct classification stage and substage. The use of primary and secondary key points on both lateral TMJ images and sagittal MRI views simplified and improved the accuracy of patient assignment. Validation of the new classification system demonstrated overall almost perfect agreement between the two radiologists.

### Patient demographic data

During the study interval, 187 patients were screened for eligibility. Six patients did not meet the inclusion criteria due to rheumatoid arthritis. Thirteen patients could not undergo MRI because of claustrophobia and were excluded. Thirteen patients had received previous TMJ treatment (repeated joint injections with dextrose prolotherapy, blood, or PRP) and were excluded. Eleven patients declined treatment after clinical and radiographic evaluation and were excluded. The final sample included 144 patients (113 females and 31 males), aged 19 to 39 years, with a mean age of 31.24 years. Table 2 presents the demographic characteristics and baseline outcome variables of each study group.

**Table 2:** Demographic features and baseline values in outcome variables within each study group.

Study Group	I-A	I-B	I-C	II-A	II-B	III-A	III-B	IV
Sample size	29 (20%)	35 (24.3%)	15 (10.4%)	25 (17.3%)	19 (13%)	7(5%)	3 (2%)	11 (8%)
Gender								
Male	6	7	7	4	3	-	-	4
Female	22	28	8	21	16	7	3	7
Mean age	30.24 ± 3.87	30.94 ± 6.38	34.87 ± 4.09	31.52 ± 3.95	30.89 ± 4.24	31.14 ± 3.34	31.00 ± 1.73	29.09 ± 5.394
MVMO	54.24 ± 3.27	54.43 ± 3.06	54.20 ± 3.28	54.64 ± 3.17	54.00 ± 2.92	53.43 ± 3.69	55.33 ± 4.51	51.73 ± 2.87
VAS	7.62 ± 0.73	7.57 ± 0.74	8.00 ± 1.07	8.08 ± 0.70	7.68 ± 0.82	8.14 ± 0.89	8.00 ± 0.0	7.36 ± 1.12
Sound	1.00 ± 00	1.00 ± 00	1.00 ± 00	1.00 ± 00	1.00 ± 00	1.00 ± 00	1.00 ± 00	1.00 ± 00

### Clinical outcome results of the treatment protocol

#### Maximum voluntary mouth opening (MVMO)

**Fig. 12:**
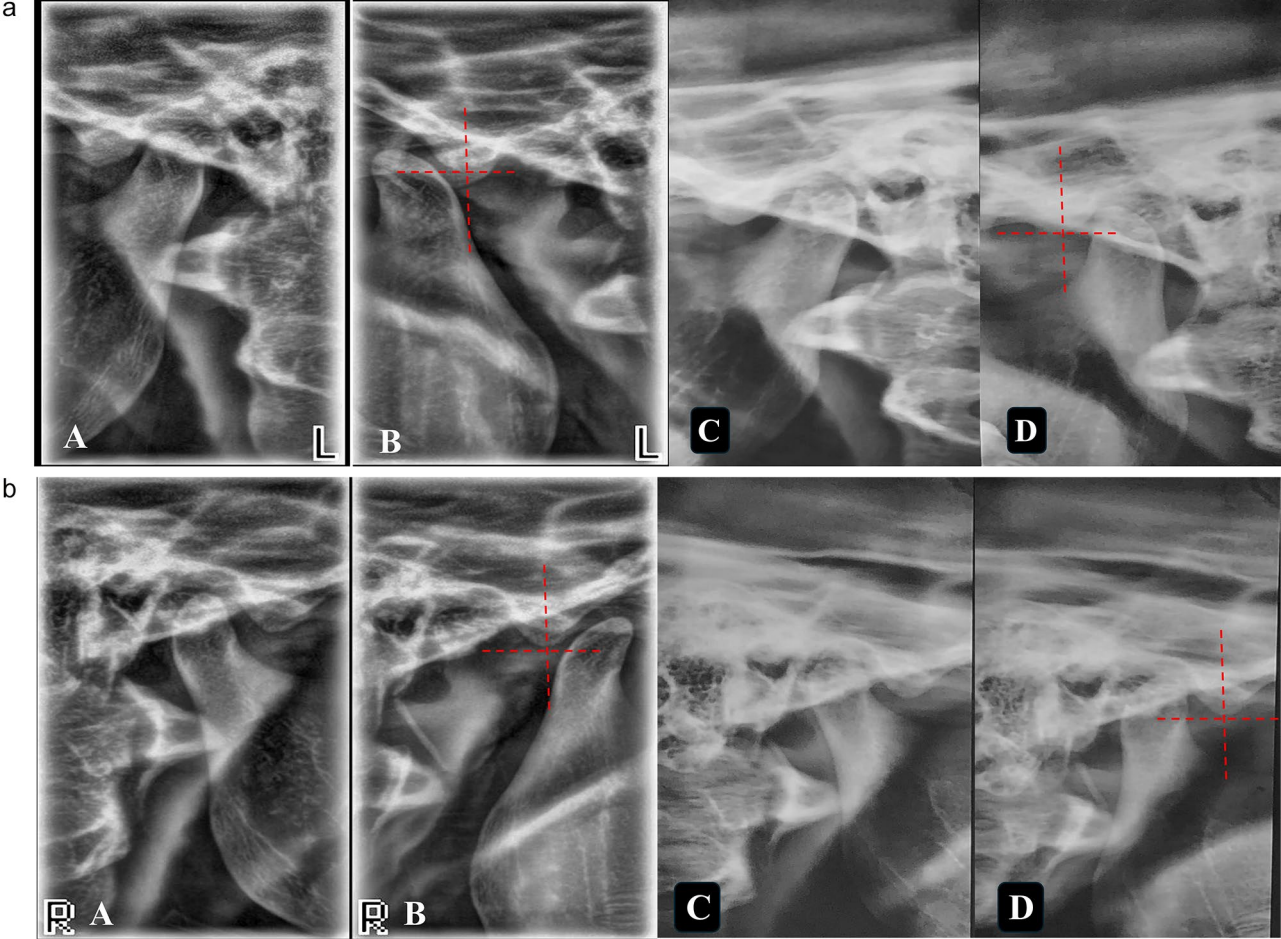
-A Left-side TMJ pretreatment in closed **(A)** and open **(B)** lateral TMJ views showed the condyle located anterior to the eminence but at the superior level of the highest point of the eminence. Posttreatment in closed **(C)** and open **(D)** lateral TMJ views showed a significant decrease in condylar translation movement.Fif. 12-B Right-side TMJ pretreatment in closed **(A)** and open **(B)** lateral TMJ views showed the condyle located anterior to the eminence but at the superior level to the highest point of the eminence. Posttreatment in closed **(C)** and open **(D)** lateral TMJ views showed a significant decrease in condylar translation movement.

Pretreatment MVMO ranged from 48 to 60 mm, with a mean of 54.13 mm. The mean MVMO decreased to 24.34 mm at one month postoperatively and reached a mean of 37.23 mm at 12 months. Statistical analysis demonstrated a significant decrease in mouth opening from pretreatment to all posttreatment time points across study groups (P < 0.0001). (Fig. 12A and B).Table 3 presents the clinical characteristics of pretreatment and posttreatment MVMO changes in all study groups across the study periods.

**Table 3:** Clinical characteristics of the different pre-treatment and post-treatment changes of the MVMO in all the study groups throughout the study periods.

MVMO	Pre-Treatment	1 M	3M	6M	12 M	P Value
Groups						
I-A	54.24 ± 3.27	24.10 ± 2.44	33.07 ± 1.56	35.07 ± 1.99	37.31 ± 1.466	< 0.0001
I-B	54.43 ± 3.06	23.91 ± 2.293	31.89 ± 1.66	33.89 ± 1.67	37.20 ± 1.389	< 0.0001
I-C	54.20 ± 3.28	24.60 ± 2.64	32.87 ± 1.77	33.60 ± 1.96	37.20 ± 1.37	< 0.0001
II-A	54.64 ± 3.17	23.75 ± 2.15	34.38 ± 1.95	35.54 ± 1.74	38.17 ± 1.01	< 0.0001
II-B	54.00 ± 2.92	25.58 ± 2.59	31.32 ± 1.53	32.74 ± 1.69	36.11 ± 1.19	< 0.0001
III-A	53.43 ± 3.69	25.14 ± 2.97	31.14 ± 3.45	34.00 ± 2.71	37.00 ± 1.41	< 0.0001
III-B	55.33 ± 4.51	24.67 ± 2.08	32.33 ± 3.79	34.67 ± 2.08	38.00 ± 1.00	< 0.0001
IV	51.73 ± 2.87	24.55 ± 2.07	32.00 ± 1.67	34.91 ± 1.87	37.27 ± 1.27	< 0.0001

#### Visual analogue scale (VAS)

Pretreatment VAS scores ranged from 6 to 9, with a mean of 7.78. The mean VAS decreased to 3.253 at one month postoperatively and reached 0.0 at six months, which was maintained through 12 months. Statistical analysis showed a significant decrease in VAS from pretreatment to all posttreatment time points across study groups (P < 0.0001).

Table 4 presents the clinical characteristics of pretreatment and posttreatment VAS changes in all study groups across the study periods.

**Table 4:** Clinical characteristics of the different pre-treatment and post-treatment changes of the VAS in all the study groups throughout the study periods.

VAS	Pre-Treatment	1 M	3M	6M	12 M	P Value
Groups						
I-A	7.62 ± 0.73	3.345 ± 0.48	0.65 ± 0.72	0.0 ± 0.0	0.0 ± 0.0	< 0.0001
I-B	7.57 ± 0.74	3.28 ± 0.86	0.54 ± 0.61	0.0 ± 0.0	0.0 ± 0.0	< 0.0001
I-C	8.00 ± 1.07	3.33 ± 0.72	0.53 ± 0.52	0.0 ± 0.0	0.0 ± 0.0	< 0.0001
II-A	8.08 ± 0.70	3.40 ± 0.50	0.60 ± 0.65	0.0 ± 0.0	0.0 ± 0.0	< 0.0001
II-B	7.68 ± 0.82	3.16 ± 0.89	0.37 ± 0.49	0.0 ± 0.0	0.0 ± 0.0	< 0.0001
III-A	8.14 ± 0.89	2.86 ± 0.38	0.28 ± 0.49	0.0 ± 0.0	0.0 ± 0.0	< 0.0001
III-B	8.00 ± 0.0	3.00 ± 0.0	0.0 ± 0.0	0.0 ± 0.0	0.0 ± 0.0	< 0.0001
IV	7.36 ± 1.12	2.91 ± 0.70	0.45 ± 0.52	0.0 ± 0.0	0.0 ± 0.0	< 0.0001

### Joint sounds

Pretreatment joint sounds were detected in all patients but completely disappeared in every patient from one month postoperatively through 12 months. Statistical analysis showed a significant reduction in joint sounds between pretreatment and all posttreatment time points across study groups (P < 0.0001).

Table 5 presents the clinical characteristics of pretreatment and posttreatment changes in joint sounds across all study groups during the study periods.

**Table 5:** Clinical characteristics of the different pre-treatment and post-treatment changes of the joint sounds in all the study groups throughout the study periods.

Joint sound	Pre-Treatment	1 M	3M	6M	12 M	P Value
Groups						
I-A	1.00 ± 00	00 ± 00	00 ± 00	00 ± 00	00 ± 00	< 0.0001
I-B	1.00 ± 00	00 ± 00	00 ± 00	00 ± 00	00 ± 00	< 0.0001
I-C	1.00 ± 00	00 ± 00	00 ± 00	00 ± 00	00 ± 00	< 0.0001
II-A	1.00 ± 00	00 ± 00	00 ± 00	00 ± 00	00 ± 00	< 0.0001
II-B	1.00 ± 00	00 ± 00	00 ± 00	00 ± 00	00 ± 00	< 0.0001
III-A	1.00 ± 00	00 ± 00	00 ± 00	00 ± 00	00 ± 00	< 0.0001
III-B	1.00 ± 00	00 ± 00	00 ± 00	00 ± 00	00 ± 00	< 0.0001
IV	1.00 ± 00	00 ± 00	00 ± 00	00 ± 00	00 ± 00	< 0.0001

### Blood injection versus I-PRF

Thirty-seven patients with TMJ osteoarthritis were treated with I-PRF, while 107 patients with joint hypermobility without osteoarthritis were treated with blood injection. At 12 months postoperatively, the mean MVMO was 39.05 mm in the I-PRF group and 36.63 mm in the blood injection group, a statistically significant difference (P < 0.0001).

Regarding VAS improvement, the mean score in the blood injection group was 3.177 compared with 3.486 in the I-PRF group. This difference was statistically significant at one month postoperatively (P = 0.009) but not significant at the end of the study (P = 0.41).

There was no statistically significant difference between the two groups in terms of improvement of joint sounds.

## Discussion

The final sample consisted of 144 patients (113 females and 31 males). Statistical analysis of the clinical outcome variables posttreatment showed a significant decrease in MVMO and pain scores (VAS) across all study groups.

Reasons for classification.

Classification is considered the process of transforming descriptions of the diagnosis of pathologic findings into universal medical codes that represent the data required for evidence-based treatment plans. Missing data will result in inadequate treatment plans, failure to achieve optimal outcomes, and potential relapse. Staging is a measure of disease severity based solely on predefined medical criteria. Classification staging makes the process replicable, easy to audit, and broadly applicable. In TMJ diseases, discrete stages can be defined and detected using MRI, reflecting the severity of the disease. These stages have clinical significance for prognosis and therapeutic decision-making.

In both classifications proposed by Akinbami and Patel et al., the position of the condylar head below the tip of the eminence was considered a degree of condylar hypermobility. During maximum normal mouth opening, however, the condylar head lies below the tip of the articular eminence with the articular disc interposed at the 12 o’clock position. This provides a clue about the validity of both classification systems^34,43,44^. The first three stages of the classification proposed by Patel et al. showed no evidence of joint hypermobility. Moreover, the classification system did not mention the state of disk position associated with hypermobility disorders.

In our classification system, we included both condylar and disk positions in cases of joint hypermobility. The condylar position was determined with the aid of lateral TMJ views, while disk position was assessed using MRI, which is considered the gold standard for evaluating disk position. MRI provides information on disk morphology and position through high soft-tissue resolution without exposing patients to ionizing radiation. It is a simple, noninvasive procedure and is now widely used worldwide. In addition, our system includes posterior disk displacement, which was not addressed in the previous classification of TMJ hypermobility.

Moreover, the new classification system is beneficial in guiding nonsurgical treatment planning with a predictable prognosis. To our knowledge, our staging system is the first hypermobility classification to demonstrate disk position in addition to the direction of disk displacement (anterior/posterior), thereby contributing to a nonsurgical treatment protocol. Our work demonstrated that, by following this classification, patients can be consistently assigned to the appropriate stage and substage.

The effectiveness of our nonsurgical treatment protocol in reducing joint pain was statistically significant in all study groups. Statistical analysis of the VAS showed a statistically significant decrease in pain scores across all study periods, with P < 0.0001 at 12 months posttreatment in all groups. Regarding MVMO, statistical analysis of pretreatment and posttreatment measurements revealed a significant decrease in all study groups across all study periods, with P < 0.0001 at 12 months. Moreover, analysis of pretreatment and posttreatment measurements demonstrated a significant decrease in joint sounds throughout the study periods in all groups (P < 0.0001). Collectively, the findings of our study suggest that the nonsurgical treatment protocol was associated with improvement in clinical outcomes across all study groups.

Application of splint thickness of 4 mm and 6 mm for cases of DDR and DDNR, respectively, resulted in anteroposterior and vertical movements of the mandibular condyle with anteroposterior movements of the articular disk, which play an important role in disk recapture^34,42^.

The application of blood injection in the treatment of mandibular hypermobility is considered a simple and safe technique and can be performed in outpatient clinics^4^.

The use of blood injection for TMJ hypermobility was first reported by Brachmann in 1964, and in 1973, Schulz was the first to report its use in treating recurrent condylar dislocation. The procedure is straightforward and does not cause foreign body reactions^45–47^.

The rationale for blood injection is that blood is injected into the pericapsular tissues and the superior joint space. Bleeding into the pericapsular tissues following needle penetration creates a bed for fibrous tissue formation. The role of injecting blood into the superior joint space is not fully understood, but it may result in the formation of intercompartmental adhesions. Importantly, restrained mandibular movement is key to the success of the procedure. In our study, jaw immobilization was achieved with an extraoral face-lift bandage, IMF screws, or orthodontic braces for 2 weeks postoperatively. Blood injection into the superior joint space and pericapsular tissues induced scarring and fibrous tissue formation, which prevented recurrent condylar dislocation^4^.

Blood injection can be repeated with minimal complications, though some concerns have been raised regarding its potential to cause ankylosis. However, this risk is unlikely because venous blood used for joint injections lacks osteoprogenitor cells, which are responsible for bone formation in cases of bony ankylosis^48^.

Exaggerated postoperative mouth opening may threaten the integrity of the fibrosis and release the limitation achieved, resulting in recurrent subluxation or dislocation. Some authors have reported that elastic bandaging is sufficient to permit the primary formation of a clot, and that post-injection pain will also restrain mandibular movements, allowing the injected blood to settle and fibrose^49,50^.

For this reason, the effects of blood injection can be improved with the aid of jaw immobilization. In the current study, the addition of jaw immobilization to blood or I-PRF injection improved the outcomes significantly.

The results of our study are in agreement with those of Hegab^4^, who conducted a prospective, randomized, controlled clinical trial for the treatment of chronic recurrent dislocation of the temporomandibular joint using autologous blood injection alone, intermaxillary fixation alone, or both together. The combined approach produced the best results among all study groups.

Platelet-rich plasma (PRP) and its second-generation form, injectable platelet-rich fibrin (I-PRF), are autologous blood products that contain high concentrations of growth factors and biologically active molecules^51^.

The clinical efficacy of PRP comes from the platelet concentrate, which contains approximately four times the normal number of cells^52^. Platelets have been shown to contain more than 30 growth factors, such as PDGF, TGF, VEGF, IGF, FGF, and EGF^53^. They also contain proteins responsible for cell adhesion and stimulation of tissue regeneration. Platelets stimulate fibroblasts to produce structural proteins involved in collagen formation, supporting remodeling and angiogenesis, and activate mesenchymal stem cells^54^. Analgesic, anti-inflammatory, and antibiotic effects have also been associated with PRP injection. It induces the production of glycosaminoglycans and can restore the level of endogenous hyaluronic acid^55,56^. Recent evidence suggests changes in the centrifugation process, which yield a higher platelet concentration and enhanced healing potential, may make I-PRF more effective than PRP^57,58^. Its quicker and easier preparation, along with the absence of aggregates, offers a promising method supported by several studies^58,59^.

The sustained release of growth factors and cytokines from liquid PRF or I-PRF at the injury site not only accelerates the resolution of inflammatory responses but also enhances overall regeneration of damaged tissues. This highlights the potential of liquid PRF/I-PRF as a biologically potent treatment option for TMD osteoarthritis. However, the clinical effectiveness of liquid PRF in managing TMDs still requires further investigation. To date, only a limited number of clinical studies have assessed the outcomes of intra-articular injection of injectable platelet-rich fibrin (I-PRF) following arthrocentesis^59,61^.

The deployment of I-PRF as a form of viscosupplementation marks a significant shift from traditional substances such as hyaluronic acid, providing not only mechanical support and lubrication but also growth factors that facilitate tissue regeneration^60^.0 This dual-action mechanism is consistent with existing literature, which suggests that the therapeutic advantages of I-PRF originate from its ability to modulate the inflammatory environment within the TMJ and promote the body’s inherent healing processes^62,63^.

The results showed that blood injection performed better than I-PRF in terms of reducing both MVMO and VAS.

We did not find any stage in which the condyle was located anterior and superior to the highest point of the eminence and associated with disc displacement without reduction. This may be explained by the presence of an irreducible disc anterior to the condyle during mouth opening, preventing the condyle from moving anteriorly and superiorly beyond the highest point of the eminence.

We used different methods of jaw immobilization. These methods were chosen based on availability. For example, some patients were undergoing orthodontic treatment and already wearing orthodontic braces, which were therefore used for immobilization. All methods had comparable performance because they were applied for a limited period^4^.

In the current study, the predominance of female patients (113 of 144) may affect the generalizability of our findings. Sex differences significantly influence TMJ hypermobility and TMDs, with women experiencing higher prevalence and potentially greater pain and dysfunction, possibly due to hormonal factors like estrogen, which can affect pain sensitivity and inflammation. These hormonal influences, together with genetic factors, may impact TMJ cartilage and joint laxity, leading to higher rates of hypermobility, clicking, and disc displacement in females, especially during reproductive years. While treatment outcomes remain under investigation, understanding these physiological differences is crucial for developing sex-specific diagnostic and management strategies for TMDs^64^.

## Conclusion

The new classification system is reasonable, reliable, and feasible, providing a detailed description of both condylar and disk positions in cases of joint hypermobility. The nonsurgical treatment protocol was simple, effective, and tailored to the specific pathological changes in the joint. Moreover, the new classification system is valuable for guiding nonsurgical treatment planning with a predictable prognosis.

## Data Availability

The datasets used and analyzed during the current study are available from the corresponding author upon reasonable request.
